# The Complex Function of Hsp70 in Metastatic Cancer

**DOI:** 10.3390/cancers6010042

**Published:** 2013-12-20

**Authors:** Kata Juhasz, Anna-Maria Lipp, Benedikt Nimmervoll, Alois Sonnleitner, Jan Hesse, Thomas Haselgruebler, Zsolt Balogi

**Affiliations:** Center for Advanced Bioanalysis GmbH, Gruberstr. 40-42, A-4020 Linz, Austria

**Keywords:** Hsp70, metastasis, invasion, trafficking, lysosome

## Abstract

Elevated expression of the inducible heat shock protein 70 (Hsp70) is known to correlate with poor prognosis in many cancers. Hsp70 confers survival advantage as well as resistance to chemotherapeutic agents, and promotes tumor cell invasion. At the same time, tumor-derived extracellular Hsp70 has been recognized as a “chaperokine”, activating antitumor immunity. In this review we discuss localization dependent functions of Hsp70 in the context of invasive cancer. Understanding the molecular principles of metastasis formation steps, as well as interactions of the tumor cells with the microenvironment and the immune system is essential for fighting metastatic cancer. Although Hsp70 has been implicated in different steps of the metastatic process, the exact mechanisms of its action remain to be explored. Known and potential functions of Hsp70 in controlling or modulating of invasion and metastasis are discussed.

## 1. Introduction

The stress-inducible heat shock protein 70 (Hsp70) also known as HSPA1A, Hsp70-1, Hsp72 or HspA1 [1], is produced at low or undetectable levels in unstressed, healthy cells. Upon a variety of stresses its expression is rapidly induced through mitogen-activated protein kinase/extracellular signal-regulated kinase (MAPK/ERK) and stress-activated protein kinase (SAPK) signaling cascades activating heat shock factors (HSFs) [2,3,4]. Hsp70 restores the balance of cell proteome by normalizing the concentration of unfolded and denatured proteins. Being a molecular chaperone, Hsp70 is an important part of cellular networks, including transcriptional, signaling, membrane and organelle networks [5].

The tumor microenvironment, where cells are subjected to free radicals, acidosis, hypoxia and nutrient deprivation, as well as high levels of mutant proteins, causes stressful conditions challenging cancer cells [6]. Accordingly, constitutive high levels of Hsp70 are frequently observed in various cancer cells [7,8], where Hsp70 enhances cell growth, suppresses senescence, and confers resistance to stress-induced apoptosis. Origin of elevated Hsp70 levels in cancer cells is thought to result from the need for antistress proteins. It has been hypothesized that elevated Hsp70 level in cancer cells is a consequence of altered HSF1 transcriptional activity [9,10,11], although Hsp70 may be also expressed regardless of HSF1 [12]. Interestingly, inhibition of Hsp70 in tumor cells is often lethal [13] and silencing of Hsp70 kills several types of cancer cells in culture as well as in tumor xenografts in mice [13,14,15]. Other rodent cancer models pointed to the tumorigenic potential of Hsp70 [16,17,18,19]. Although a large body of evidence supports the importance of Hsp70 in oncogenesis, the exact mechanisms remain elusive.

Expression level of Hsp70 is a diagnostic measure in several cancers, as Hsp70 overexpression can be correlated with increased cancer cell proliferation [20], clinical stage [21,22] or increased grade and shorter overall survival [23]. Extensive research in the last decades potentiated Hsp70 as a marker molecule in cancer treatment. Hsp70 is a good tumor marker to identify patients with early-stage prostate cancer [24] and hepatocellular carcinoma [25]. High expression levels of Hsp70 correlate with poor prognosis in acute myeloid leukemia, in cancers of the breast, endometrium [8,26,27,28] and rectum [29]. Furthermore, Hsp70 expression might be of use to assess the progression of esophageal squamous cell carcinoma [30,31]. However, elevated Hsp70 level is not a general marker of poor prognosis, as it has no prognostic relevance in gastric cancer [29,32], or even indicates good prognosis in renal and esophageal cancer [7,33]. Interestingly, Hsp70 levels correlate with malignancy in osteosarcoma and renal cell tumors, whereas associate with improved prognosis [7,34]. Accordingly, association of Hsp70 expression and clinical outcome largely depends on the cancer type and stress conditions. Cancer cell specific surface localization or release of Hsp70 exhibits additional activities of this stress protein [35,36,37,38,39]. Hsp70 exerts a dual role in cancer, promoting survival and dissemination of tumor cells, and at the same time contributing to antitumor immunity.

Metastasis is a result of a series of highly orchestrated processes, including epithelial-mesenchymal transition (EMT), alteration of cell adhesion and motility, inducing neoangiogenesis, invasion into tissue, intravasation, and surviving in the blood or lymphatic vessels. Besides the extensively studied Hsp90, Hsp70 family members have been implicated in metastasis formation as well [40,41,42]. Elevated Hsp70 expression has been found to correlate with lymph node metastases and decreased survival in breast cancer models [43]. It has been hypothesized that membrane Hsp70, like membrane Hsp90 [44], might support the spread of distant metastasis. The fact that Hsp70 expression can influence metastasis development and drug resistance further highlights the need for understanding its role in cancer progress [45,46,47]. This review focuses on our current understanding of the pleiotropic properties of Hsp70 in metastatic cancer cells.

## 2. Hsp70 Supports Metastatic Cancer Cell Growth through Chaperone and Antiapoptotic Functions

Elevated Hsp70 expression, frequently associated with transformed phenotype, may provide a selection advantage to cancer cells, whereas depletion of Hsp70 promotes G2/M cell cycle arrest [47] and tumor regression [19]. It has been assumed that elevated Hsp70 expression relates to cell growth in epithelial carcinoma cell lines [48,49]. Hsp70 as a molecular chaperone has long been in the focus of cancer research that revealed a number of client proteins interacting with Hsp70 during cell growth (reviewed in [50]). Among several hypotheses on the role of Hsp70 in human malignancies it has been suggested that high levels of inducible Hsp70 in tumor cells may be required for stabilizing mutant oncogene products during tumor growth [51,52]. Detailed molecular mechanisms of chaperone activity of Hsp70 enhancing tumor cell growth have been reviewed elsewhere [53].

Because of shear stress through vasculature transit and lacking of survival signals from adhesive sites, metastatic steps are particularly stressful for disseminating cells, making metastasis an ineffective process [54,55]. Therefore, survival pathways must be enhanced in order for selection of aggressive tumor cells. Metastatic cells likely benefit from Hsp70 chaperone functions supporting cellular growth. Nevertheless, the beneficial effect of Hsp70 on cell growth widely reported for primary cancer cells remains largely unexplored for metastatic cells. In fact, it is mainly coupled to other aspects of metastasis, such as cytoskeleton-dependent signaling. Indeed, proliferation of human colonic carcinoma cells correlated with co-expression of Hsp70 and CD44 [56], a surface molecule implicated in tumor cell survival during micrometastasis formation [57].

Besides chaperoning cancer cell growth, Hsp70 has been shown to inhibit cancer cell death induced by different stimuli such as oxidative stress, inflammatory cytokines, anticancer drugs or irradiation [58,59,60,61,62,63,64,65]. Consistently with its known substrate promiscuity, Hsp70 interacts with multiple partners in the signaling cascades of apoptosis and senescence. Hsp70 has been shown to directly bind to and inhibit apoptosis signal-regulated kinase 1 (Ask1), p38 MAPK and c-Jun N terminal kinase (JNK), thereby blocking stress-induced cell death [66,67,68,69]. Hsp70 modulates ERK signaling upon heat stress and hyperosmolarity-induced apoptosis [70,71,72]. In tumor cells, there has been shown a suppressive role of Hsp70 in senescence through controlling p53 and ERK activity [45,73]. Inducible Hsp70 plays a negative regulatory role in the mitochondrial apoptotic pathway at several steps. Hsp70 directly binds to apoptosis protease-activating factor-1 (Apaf-1), thereby preventing the recruitment of procaspase-9 to the apoptosome [74]. Hsp70 interferes also with caspase-independent apoptotic pathways, interacting with apoptosis-inducing factor (AIF), in turn inhibiting AIF-induced chromatin condensation [75]. Additionally, Hsp70 has been shown to accumulate in the lysosomes of many tumor cell types, preventing lysosomal membrane permeabilization-induced cell death [65,76]. Detailed mechanisms of the involvement of Hsp70 in apoptotic pathways are reviewed elsewhere [9,53,77].

Avoiding apoptosis is crucial for tumor cells during the metastatic process. Alteration in cell adhesion to extracellular matrix (ECM) proteins is one of the earliest steps in cancer metastasis [78]. Involvement of signaling molecules such as focal adhesion kinase (FAK), Met and Akt have been shown to play a role in anoikis and amorphosis, apoptotic processes that normally occur to cells losing contact with ECM [79]. Activity of these molecules has been reported to be influenced by Hsp70 [80,81,82], raising a possible regulatory role of Hsp70 in these processes typically inhibited in metastatic cancer cells. It has also been suggested that Hsp70 could directly protect members of cytoskeletal-based cell survival pathways [14]. Indeed, interaction between FAK and Hsp70 prevents FAK degradation by activated caspase-3 and may represent a novel mechanism for cytoprotection [82]. It has been recently shown that matrix metalloproteases (MMPs), in addition to being active during invasion, are involved in regulating apoptosis [83,84]. MMPs may interfere with cell death by cleaving death receptors, as well [85]. Since Hsp70 expression is known to affect MMP secretion and activity in cancer [86,87], Hsp70 levels may indirectly determine the fate of a metastatic cancer cell. Further studies using appropriate models would be still necessary to dissect the role of Hsp70 in conferring a survival advantage on cancer cells at different steps of metastasis.

## 3. Hsp70 Supports Metastasis through Promoting Invasion Steps

Upon tumor progression and antitumor treatments cancer cells are exposed to various forms of stress such as proteasome inhibition, hypoxia or heat stress, which have been reported to induce metastatic steps including EMT, cell migration and invasion [88,89]. Hsp70 overexpression correlates with a more aggressive phenotype in several cancer types. Elevated expression of Hsp70 has been found to correlate with lymph node metastasis in breast cancer cells as mentioned above [43] and with vascular invasion in gastric cancers [90]. In cervical and bladder cancer cells shRNA knockdown of Hsp70 has been shown to suppress invasion and migration [91]. In accordance, Rohde *et al*. suggested a role for Hsp70 in cancer cell adhesion, as depletion of Hsp70 resulted in cell detachment [47].

During invasion tumor cells need to penetrate surrounding tissue. This process requires enhanced cell motility and reduced adhesion, acquired by EMT. Notably, the carboxyl-terminus of Hsp70 Interacting Protein (CHIP) downregulates Met, one of the key receptors triggering EMT [92] via a switch from Hsp70 chaperone activity to proteosomal targeting [81]. Essential role of Hsp70 in transforming growth factor-beta (TGF-beta) induced EMT has been revealed, where Hsp70 blocks TGF-beta signaling by impeding Smad2 phosphorylation [93]. As EMT is thought to be a prerequisite for the invasive behavior of cancer cells, further studies may help targeting Hsp70 to prevent metastasis. Although Hsp70 is known to influence the activity of molecules involved in cell motility [41,82,94], little is known about the role of Hsp70 in cancer cell migration. Knockdown of Hop caused a decrease in the level of RhoC GTPase, and significantly inhibited pseudopodia formation in cancer cell lines [95]. Activity of Hsp70/Hsc70 has been found essential for cell motility revealed by experiments inhibiting their ATPase activity in EGF stimulated cells [96]. In the same publication tissue transglutaminase was identified to translocate to the leading edge of the cell depending on active Hsp70. Remarkably, at the same site further interaction partners of Hsp70 are present that are involved in cell motility. Hsp70 is known to stabilize FAK [82] as well as Wiskott-Aldrich syndrome protein family member 3 (Wasf3) [91], a molecule involved in lamellopodia formation and metastasis [97,98,99]. A potential linker represents the Hsp70 regulator BAG3. The SH3 binding motifs of BAG3 could target Hsp70 protein complexes to signaling complexes at the leading edge of the cell. Indeed, reduced motility of BAG3-deficient mouse endothelial fibroblasts and BAG3 depleted cancer cell lines was observed [100]. In addition, BAG3 provides a potential link to the modulation of the extracellular matrix, another key step in invasion. Immunoprecipition indicated BAG3 as an interaction partner of MMP-2 [101].

**Figure 1:**
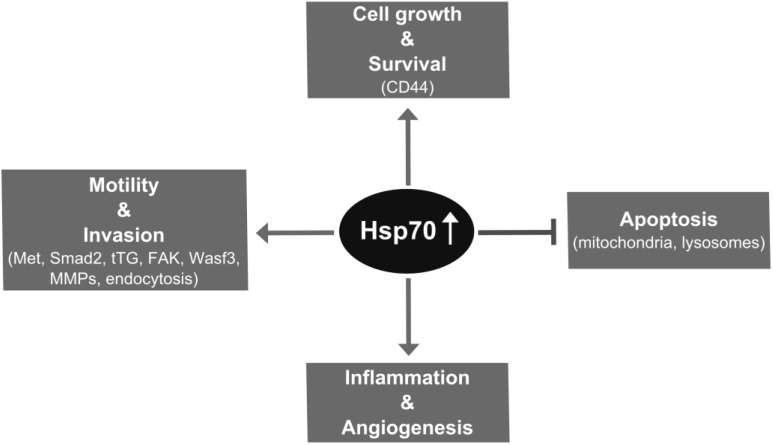
Potential metastasis promoting activities of Hsp70. Elevated expression of Hsp70 in tumor cells has beneficial effect on metastatic cell growth through cytoskeleton-dependent signaling. Hsp70 interacts with multiple partners in the mitochondrial and lysosomal signaling cascades of apoptosis, providing selection advantage for aggressive tumor cells by antiapoptotic activities. Hsp70 enhances motility and invasion through interactions with proteins involved in EMT, lamellipodia formation and ECM degradation. Tumor-derived Hsp70 promotes inflammatory conditions in the tumor microenvironment, thereby enhancing invasion and angiogenesis, in turn metastasis.

Besides the rather indirect link of Hsp70 to MMPs via BAG3, MMP-2 is directly activated by Hsp90 [102] in an extracellular chaperonin complex with Hsp70/Hsp90 organizing protein (Hop) and Hsp70 [103]. Knockdown of Hop reduced the expression of MMP-2 and other proteins implicated in invasion and metastasis [103]. Whether MMP levels correlate with negative outcome and tumor aggressiveness is still unclear [104]. Remarkably, in glioblastoma xenografts CD44, a receptor involved in EMT and migration [105] is activated by MMP-9 [106], which in turn, is released in the presence of extracellular Hsp70 [87]. Furthermore, the presence of extracellular Hsp70 can trigger an inflammatory microenvironment and angiogenesis, which are hallmarks of cancer development as discussed later in this review. Therefore, Hsp70 has multiple activities which potentially contribute to invasion and metastasis (Figure 1).

## 4. Impact of Hsp70 Trafficking on Metastasis

Increased level of Hsp70 in patients’ blood samples and in the extracellular milieu appears to be a general feature of cancers [107]. Tumor-derived extracellular Hsp70 can be attributed to necrotic cell death, where the large amount of mainly intracellular chaperone signals as danger for the immune system. In addition, active release mechanisms have been reported as source of extracellular Hsp70, depending on cell type and conditions. The exact mechanism of tumor-specific surface targeting and release of Hsp70, as well as mechanistic details of its intracellular trafficking still remain to be explored.

Hsp70, a leaderless chaperone has been proposed to cross the membrane through its ability to bind to phosphatidylserine [108] and formation of pores shown in artificial lipid membranes [109]. The authors suggested a mechanism of surface expression and release independent of vesicular trafficking. Association of Hsp70 with lipid rafts has also been reported [110], which was consistent with the idea of some involvement of membrane domains in active release of Hsp70 [110,111,112]. The endolysosomal system has been implicated in Hsp70 trafficking in various cell types [37,65,110,112,113,114]. Exosomal transport of Hsp70 has been shown for tumor cells *in vitro* and *in vivo*, which could be facilitated by drugs or heat shock [115,116,117]. In addition, in human peripheral blood mononuclear cells, “exonemes” have been identified to secrete Hsp70 [113]. As an alternative mechanism, Hsp70 has been shown to be actively released via secretory lysosomes in a soluble form [114,118]. Interestingly, excess of Hsp70 in a melanoma model was shown to facilitate lysosomal routing, surface expression and release of Hsp70 [118]. Noteworthy, internalized surface Hsp70 has been demonstrated to be released in both membrane-bound and soluble forms [37,118]. Removal of internalized surface Hsp70 that trafficked through the endosomal/lysosomal sytem has been suggested as potentially highly immunogenic.

In fact, a subfraction of Hsp70 has been found to accumulate in lysosomes of many cancer cell types. Hsp70 has been also demonstrated to bind to the limiting membrane of lysosomes at the lumenal side, through pH-dependent high-affinity binding to bis(monoacylglycero)phosphate (BMP) [119]. Hsp70 has been suggested to be targeted to lysosomes by autophagy [120,121] or by endocytosis from the plasma membrane [122], unexplored proposed mechanisms of Hsp70 membrane crossing. Interestingly, Hsp70 appears to inhibit a unique pathway of cell death in tumor cells, which involves lysosomal membrane permeabilization and activation of caspase-3 [65,123]. Hsp70 localized to membranes of the endosomal/lysosomal compartment counteracts lysosomal membrane permeablization and release of cathepsins, in turn preventing apoptosis [65,80].

**Figure 2:**
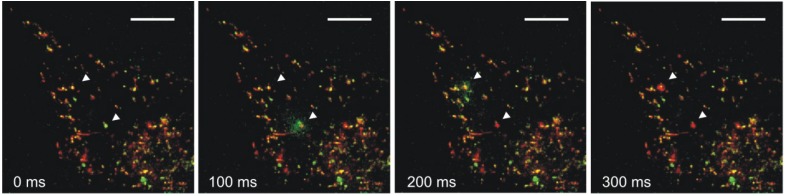
Lysosomes secreting Hsp70 from melanoma cells. Stable clones of mouse B16 cells were generated and induced to express Hsp70-E3. Hsp70-E3 exposed at the cell surface was labelled with the complement peptide K4 conjugated to AlexaFluor488 [128]. Prior to imaging, cells were transiently transfected with LAMP-1-mKate to label the late endosomal/lysosomal membrane. Release of Hsp70 via lysosomes fusing with the cellular membrane was visualized by total internal reflection fluorescence (TIRF) microscopy upon addition of 1.4 µM ionomycin (scale bar = 10 µm). Note that release of the Hsp70-E3-K4 adduct could also be triggered by heat stress [118,127] Hsp70 and LAMP-1 are displayed in green and red, respectively. Arrowheads indicate sites of vesicular fusion. Note shedding the soluble lysosomal content (Hsp70) followed by spreading of the vesicular membrane marker (LAMP-1) in the plasma membrane. See also movie as supplementary information.

In addition to protecting cells from apoptosis through lysosomal membrane stabilization [65], Hsp70 sustains the activity of acid sphingomyelinase (ASM). Hsp70 and ASM have been proposed to influence dynamics of the vesicular system, including endosomes, multivesicular bodies, autophagosomes and lysosomes. Moreover, it has been hypothesized that Hsp70 may regulate trafficking of these organelles [119]. Proper trafficking of lysosomes may be crucial for tumor growth and metastasis formation, as enhanced release of lysosomal content into the extracellular space can facilitate matrix degradation, in turn invasion [124]. Indeed, trafficking of lysosomes appears to be altered in cancer cells. As compared to healthy cells, cancer cells have more lysosomes near the cell surface, which more frequently fuse with the plasma membrane and secrete their content such as cathepsins capable of promoting invasion and angiogenesis [125,126]. In support of the idea that Hsp70 may influence lysosomal trafficking, we observed Hsp70 to accumulate in the lysosomes of Hsp70 overexpressing melanoma cells, which correlated with an increased rate of lysosomal trafficking measured as lysosomal-associated membrane protein-1 (LAMP-1) surface exposure [127]. Moreover, we also visualized Hsp70 being released from lysosomes that fuse with the plasma membrane upon different stress triggers (Figure 2).

This potentiates that Hsp70 may indirectly or directly influence secretion of lysosomal enzymes digesting the extracellular matrix, hence facilitating tumor cell invasion. Nevertheless, such potential role, and in particular mechanism of Hsp70 action in regulation of lysosome trafficking remains to be explored. This task would require appropriate tools enabling to monitor intracellular trafficking during invasion. Spatiotemporal visualization of cancer cells during invasion at the molecular level sets technically challenging requirements. Cells need to be monitored in real time, in multiple focal planes with high temporal and spatial resolution in tissue or extracellular matrix material. Future technical developments, therefore, may help to get a deeper insight into localization dependent function of Hsp70 in a complex and dynamic environment.

Hsp70 has been implicated in endosomal trafficking of cancer cells. Elevated expression of Hsp70 gave rise to trafficking of CD44v6 to the plasma membrane [129] or clathrin dependent endocytosis measured by transferrin uptake in human hepatoblastoma cells [122]. Enhanced endocytosis of nutrients may also serve as a pro-cancer activity of Hsp70 expression [129]. Hsp70 is capable of supporting or counteracting cancer progression, dependent on the fate of an individual tumor cell. Notably, exposure of the immune stimulatory Hsp70 on the tumor cell surface has been considered as an “unfortunate consequence” of lysosomal release mechanisms, which may explain why tumor cells with surface Hsp70 positivity and Hsp70 release are not selected during carcinogenesis and metastasis [80]. Therefore, although avoiding elevated extracellular Hsp70 may be a favorable approach in anticancer therapies, surface Hsp70 positivity of survival cancer cells stressed by therapy may account for a therapeutic advantage if an enhanced immune response against the resistant populations is achieved. The importance of surface Hsp70 expression and signaling as well as coordinated vesicle trafficking in invasion and metastasis have been only recently recognized [107]. Further studies on the influence of Hsp70 on vesicular trafficking may contribute to control cancer cell invasion.

## 5. Extracellular Functions of Hsp70

**Figure 3:**
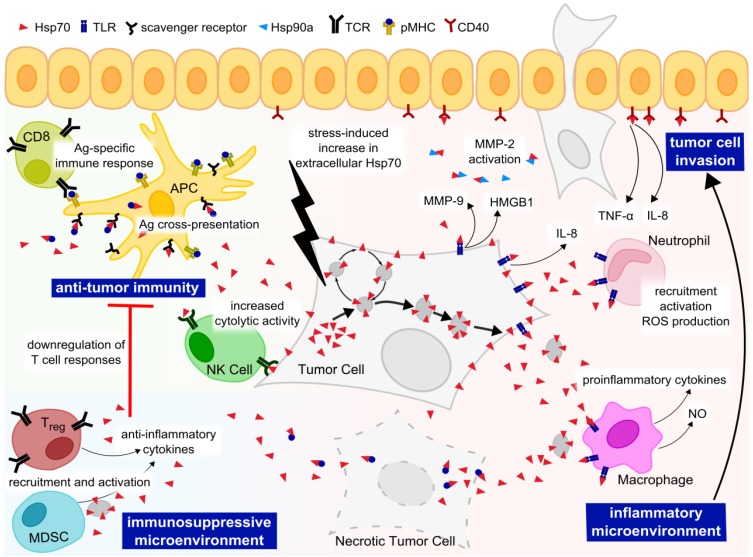
Versatile functions of extracellular Hsp70. Extracellular Hsp70 is able to interact with receptors expressed on cells of the tumor microenvironment. Hsp70 complexed to tumor-derived peptide binds to scavenger receptors on antigen presenting cells (APC), and is internalized and cross-presented to CD8^+^ T cells, thereby an adaptive tumor-specific immune response is initiated. Stimulation of NK cells by Hsp70 leads to increased cytotoxic activity against Hsp70-positive tumor cells. Hsp70 has an immunosuppressive role via recruitment and activation of regulatory T cells (Treg) and myeloid-derived suppressor cells (MDSC), leading to the downregulation of T cell responses. Hsp70 acts as a danger signal via binding to Toll-like receptors (TLRs) on mononuclear cells, leading to secretion of pro-inflammatory cytokines and nitric oxide (NO), in turn providing an inflammatory environment that contributes to metastasis formation. Hsp70 promotes tumor invasion and angiogenesis through activation of MMP-2 and production of ROS by neutrophils, respectively. Binding of Hsp70 to epithelial cells results in secretion of pro-inflammatory cytokines, activating an amplification loop.

Cancer-specific surface expression and release of Hsp70 further increase the already broad spectra of Hsp70 activities. Depending on the release mechanism, extracellular Hsp70 exists in a free soluble form, complexed to antigenic peptides, or in exosomes [37,110,113,130]. It seems likely that different forms of extracellular Hsp70 mediate distinct functions, mainly through interactions with different types of target cells and subsequent signaling. The observed signaling capacity of extracellular Hsp70 can be ascribed to its interaction with a number of transmembrane immune receptors differentially expressed on cells in the tumor microenvironment, including immune cells, tumor cells, endothelial and epithelial cells (Figure 3).

Extracellular Hsp70 complexed to tumor peptides has been shown to interact with scavenger receptors on antigen-presenting cells, like CD91, SREC-1, and LOX-1 [131,132,133,134]. This is followed by peptide-Hsp70 complex uptake via receptor-mediated endocytosis, leading to antigen cross-presentation on MHC I molecules [135,136,137,138] and an adaptive tumor-specific immune response mediated by CD8^+^ cytotoxic T cells [136,138,139,140]. Hsp70 of tumor-derived exosomes has been reported to activate natural killer (NK) cells [37]. In accordance, treatment with full-length Hsp70 or the 14-mer peptide TKD, being identified as the fragment of Hsp70 exposed on the tumor cell surface [141], triggers expression of activating receptors, such as CD94, and initiates proliferation, cytolytic and migratory capacity of resting NK cells [37,142,143,144]. Similar effects upon Hsp70 exposure have been described for CD4^+^ T helper cells, as well [145]. Noteworthy, enhanced immune functions of exosomes appear to be associated with Hsp70 expressed in the exosomal membrane [146].

Peptide-free extracellular Hsp70 has been reported to act on the innate immune system, predominantly via activation of Toll-like receptor (TLR) 2/4 signaling [39,147,148]. It has been shown that exposure to extracellular Hsp70 results in the release of proinflammatory cytokines and nitric oxide (NO) from macrophages [39,130,149,150,151], as well as upregulation of costimulatory molecules, chemoattraction and activation of dendritic cells (DCs) [149,152,153,154]. In this way, extracellular Hsp70 acts as a danger signal and provides an inflammatory tumor microenvironment. However, since Hsp70 can bind LPS, which also triggers TLR4 signaling, these data have to be interpreted carefully regarding possible effects mediated by LPS contamination rather than Hsp70 itself, as reviewed elsewhere [155,156,157,158,159].

Cancer-related inflammation plays a pivotal role in cancer development. Infiltrating immune cells might exert antitumor activity at early stages, but support tumor growth and metastasis at a chronic stage (reviewed in [160]), suggesting a role for extracellular Hsp70 in tumor progression. Additionally, extracellular Hsp70 could contribute to tumor progression via promoting an immunosuppressive tumor microenvironment, as well. It has been shown that free or exosome-bound Hsp70 can recruit and activate Foxp3^+^ regulatory T cells [161] or myeloid-derived suppressor cells, respectively [162]. This immunosuppressive tumor microenvironment eventually results in production of anti-inflammatory cytokines and dampened T cell responses through suppression of T cell proliferation and induction of tolerogenic DCs [161,163,164,165]. Recent data in mice and human models showed that tumor-derived exosomes exposing Hsp70 on the surface impair anti-tumor immunity [162].

Besides acting on immune cells, extracellular Hsp70 can trigger signaling in tumor cells in an autocrine or paracrine fashion via binding to TLR2/4, thereby playing a role in invasion and angiogenesis. It has been shown that extracellular Hsp70 released from heat-stressed A431 squamous carcinoma cells triggers autocrine epidermal growth factor receptor (EGFR) and MAPK signaling via TLR2/4 [166]. EGFR signaling is involved in MMP activation [167] and secretion of interleukin (IL)-8 [168], and has often been associated with tumor invasion and metastasis [169]. In another study binding of extracellular Hsp70 to TLR2/4 on H22 hepatocarcinoma cells triggered nuclear factor kappa-light-chain-enhancer of activated B cells (NF-κB) pathways, resulting in proliferation and resistance to apoptosis [170]. Tumor progression mediated by extracellular Hsp70 was further enhanced via a positive feedback loop stimulating a delayed activation of the JNK signaling cascade, leading to release of high mobility group protein B 1 (HMGB1) and upregulation of MMP-9 [171], both molecules playing a major role in tumor growth and invasion [172]. Similarly, extracellular Hsp70 stimulated NF-kB/AP-1 signaling increased phorbol 12-myristate 13-acetate (PMA) induced activation of MMP-9 transcription in human mononuclear cells and led to enhanced invasiveness *in vitro* [87]. More recent data show that extracellular Hsp70 secreted from breast cancer cells could form an extracellular complex with chaperones, including Hsp90a [86]. This co-chaperone complex increased binding of Hsp90a to MMP-2, and subsequent activation of MMP-2 *in vitro* [86]. Inhibiting Hsp70 in conditioned media reduced MMP-2 activation and decreased breast cancer cell migration and invasion *in vitro*, highlighting a receptor-independent role for Hsp70 in tumor cell invasion [86].

Hsp70 could increase tumor cell invasiveness through its ability to trigger an inflammatory tumor microenvironment. Besides activating antigen-presenting cells (APCs), free extracellular Hsp70 released from ovarian cancer cells has been shown to activate neutrophils and subsequent reactive oxygen species (ROS) production via TLR2/4 signaling [173]. ROS promote angiogenesis and metastasis via stimulation of vascular endothelial growth factor production [174] and activation of MMPs [175]. This neutrophil-mediated angiogenesis could be further enhanced via interplay of extracellular Hsp70 with epithelial cells. In human bronchial epithelial cells extracellular Hsp70 has been shown to induce IL-8 and tumor necrosis factor (TNF) production, leading to the attraction and activation of neutrophils [176]. Hsp70 has been reported to bind to various epithelial and endothelial cell lines [132]. Specifically, extracellular human and mycobacterial Hsp70 has been shown to bind to the TNF receptor family member CD40 [177,178], and the binding of human Hsp70 to CD40 led to its internalization and p38 signaling in CD40-expressing HEK293T cells [177]. Interestingly, epithelial and endothelial cells have also been reported to express CD40 on their cell surface, which was upregulated at inflammatory conditions [179]. Futagami *et al.* showed that treatment of HUVEC cells with extracellular Hsp70 blocks CD40L-mediated inhibition of apoptosis, as well as CD40L-induced tubular formation *in vitro*, suggesting a critical role in tumor progression and invasion [180]. Interestingly, Hsp70 does not only act on endothelial cells, but was also shown to be released from rat arterial endothelial cells in an exosome-associated form, and release was further enhanced upon oxidative stress [181]. Therefore, Hsp70 released from both tumor cells and endothelial cells might provide an autocrine and paracrine regulation mechanism for promoting tumor cell invasion via stimulating release of inflammatory mediators from bystander immune cells and secretion of MMPs and IL-8 from tumor cells. Interestingly, re-invasion into the new host tissue has recently been shown to be supported by bone marrow derived cells (BMDCs) reprogrammed by TRYP2, VLA4, Hsp70, Met and Rab27a positive exosomes released from primary cancer. The exosomes elevated Met expression and provasculogenic phenotype of the BMDCs and translocation to the lung and lymph nodes, where these cells could aid angiogenesis, invasion and metastasis [182]. 

## 6. Targeting Hsp70 in Metastatic Cancer

Versatile functions of Hsp70 are reflected in diverse approaches which have evolved for Hsp70-based anticancer therapy, including inhibition of activity, modifying of expression levels and antitumor vaccines, extensively reviewed elsewhere [183,184,185]. As knock-down of Hsp70 in cervical, bladder, breast and endometrial cancer cell lines has been proven to reduce invasiveness *in vitro* [40,91,186,187], Hsp70-specific inhibitors could be promising in prevention of invasiveness and metastasis, as well. However, there are only a few approaches specifically targeting cells with metastatic potential through Hsp70, even though this holds true for most molecular targets in recent anticancer therapy [188].

A novel Hsp70-based vaccine has been developed to selectively eliminate highly metastatic cancer stem cells. Hsp70 complexed to tumor antigens was isolated from fusions of DC and radioresistant mammary tumor cells (Hsp70.PC-F). Treatment of mice with the Hsp70.PC-F vaccine stimulated a tumor-specific CTL response against primary and disseminated tumor and rendered the tumor more sensitive to radiotherapy [189]. This approach has a high potential in working synergistically with radiotherapy to efficiently fight primary and metastasizing tumors. Another possibility to target metastasis directly is adoptive NK cell therapy in patients with Hsp70 membrane positive tumors. It has been shown that Hsp70 membrane positive tumors are targets for NK cells *in vivo* [36,190,191,192,193,194]. Moreover, NK cells stimulated *ex vivo* with TKD/IL-2 have been successfully used in a Phase I clinical trial in patients with colorectal and lung carcinoma [195,196]. Importantly, metastatic tumors have been found to express high levels of surface Hsp70 [192,197]. These characteristics are beneficial in immunotherapy using TKD/IL-2 stimulated NK cells in combination with conventional treatments and may hold promise for eliminating metastasis in patients with Hsp70 membrane positive tumors.

Extracellular Hsp70 can act in an autocrine and paracrine fashion on tumor, immune and endothelial/epithelial cells to promote tumor cell invasion and metastasis by enhancing inflammation, angiogenesis, tumor growth, and recruitment of immunosuppressive immune cell. Therefore, targeting extracellular Hsp70 through neutralizing antibodies or small molecules interfering with Hsp70 binding to receptors may be useful to prevent invasiveness of tumors. However, the benefits of extracellular Hsp70 from the patient’s point of view should be also considered in these cases. Modulating localization or activity of extracellular Hsp70 in therapeutic approaches may also help to limit cancer metastasis. A better understanding of the role of Hsp70 in tumor progression may support development of specific invasion preventing approaches.

## 7. Concluding Remarks

Elevated expression, endosomal/lysosomal localization as well as surface exposure and release represent cancer specific features of Hsp70. A yet unresolved regulation of Hsp70 activity enables diverse functions of this molecular chaperone, affected by its localization and interacting partners. Common and unusual activities of Hsp70 may also be dependent on post-translational modifications and the actual cellular environment. Hsp70 surrounded by the tumor microenvironment has been reported to influence metastatic activity positively and negatively. Therefore, targeting of Hsp70 necessitates careful considerations in cancer therapy. While Hsp70-based vaccines and NK cell therapies would make use of antitumor properties of extracellular Hsp70, targeting intracellular Hsp70 by inhibitors in conventional anticancer treatments generally attacks pro-tumor and antitumor activities of Hsp70. Increasing immunogenicity of cancer cells that are resistant to conventional therapies could be another strategy for fighting metastatic cancer. Controlled trafficking to the tumor cell surface or release of Hsp70 may be used to stimulate the patient’s immunity against the aggressive cancer cell population. For this, further investigations on Hsp70 trafficking and release, as well as on the cross-talk between tumor cells, immune cells, endothelial and epithelial cells mediated by extracellular Hsp70 in metastasis will be required. Understanding and controlling the complex function of Hsp70 in metastasizing cells will certainly require developing *in vitro* approaches and animal models of human cancers. Novel *in vitro* 3D models of invasion, enabling spatiotemporal multiparameter testing, may help further revealing the molecular mechanism of metastasis formation. Recently developed “Organs-on-Chips”, described also as 3D organs grown in microfluidic chips hold promise for real time monitoring of cellular processes during metastasis.

## Abbreviations

AIF: apoptosis-inducing factor
Apaf-1: apoptotic protease activating factor-1
APCs: antigen-presenting cells
Ask-1: apoptosis signal-regulated kinase 1
ASM: acid sphingomyelinase
BMDCs: bone marrow-derived cells
BMP: bis(monoacylglycero)-phosphate
CHIP: carboxyl-terminus of Hsp70 interacting protein
DCs: dendritic cells
ECM: extracellular matrix
EGFR: epidermal growth factor receptor
EMT: epithelial-mesenchymal transition
ERK: extracellular signal-regulated kinase
FAK: focal adhesion kinase
HMGB1: high mobility group protein B1
Hop: Hsp70/Hsp90 organizing protein
HSF: heat shock factor
Hsp70: heat shock protein 70
HspBP1: Hsp70 binding protein 1
JNK: c-Jun *N*-terminal kinase
LAMP-1: lysosomal-associated membrane protein-1
MAPK: mitogen-activated protein kinase
MDSC: myeloid-derived suppressor cells
MMPs: matrix metalloproteases
NF-κB: nuclear factor kappa-light-chain-enhancer of activated B cells
NK: natural killer
NO: nitric oxide
PMA: phorbol 12-myristate 13-acetate
pMHC: peptide-loaded major histocompatibility complex
ROS: reactive oxygen species
TCR: T cell receptor
TGF-beta: transforming growth factor-beta
tTG: tissue transglutaminase
TLR: Toll-like receptor
TNF: tumor necrosis factor
Wasf3: Wiskott-Aldrich syndrome protein family member 3

## Supplementary Information

Movie 1 Lysosomes secreting Hsp70 from mouse B16 melanoma cells. Release of Hsp70 via lysosomes fusing with the cellular membrane was visualized by TIRF microscopy. Hsp70 and LAMP-1 are displayed in green and red, respectively. The movie is playing 5 fps. Note shedding the soluble lysosomal content (Hsp70) followed by spreading of the vesicular membrane marker (LAMP-1) in the plasma membrane. Supplementary (AVI, 540 KB)
